# Membrane Remodeling by the Double-Barrel Scaffolding Protein of Poxvirus

**DOI:** 10.1371/journal.ppat.1002239

**Published:** 2011-09-08

**Authors:** Jae-Kyung Hyun, Cathy Accurso, Marcel Hijnen, Philipp Schult, Anne Pettikiriarachchi, Alok K. Mitra, Fasséli Coulibaly

**Affiliations:** 1 School of Biological Sciences, the University of Auckland, Auckland, New Zealand; 2 School of Biomedical Sciences, Monash University, Clayton, Australia; Institut Pasteur, France

## Abstract

In contrast to most enveloped viruses, poxviruses produce infectious particles that do not acquire their internal lipid membrane by budding through cellular compartments. Instead, poxvirus immature particles are generated from atypical crescent-shaped precursors whose architecture and composition remain contentious. Here we describe the 2.6 Å crystal structure of vaccinia virus D13, a key structural component of the outer scaffold of viral crescents. D13 folds into two jellyrolls decorated by a head domain of novel fold. It assembles into trimers that are homologous to the double-barrel capsid proteins of adenovirus and lipid-containing icosahedral viruses. We show that, when tethered onto artificial membranes, D13 forms a honeycomb lattice and assembly products structurally similar to the viral crescents and immature particles. The architecture of the D13 honeycomb lattice and the lipid-remodeling abilities of D13 support a model of assembly that exhibits similarities with the giant mimivirus. Overall, these findings establish that the first committed step of poxvirus morphogenesis utilizes an ancestral lipid-remodeling strategy common to icosahedral DNA viruses infecting all kingdoms of life. Furthermore, D13 is the target of rifampicin and its structure will aid the development of poxvirus assembly inhibitors.

## Author Summary

Poxviruses are arguably the largest viruses infecting humans. The unique brick-shape architecture of their infectious virus particles sets them apart from any other viral family in the virosphere. The infectious particles are produced through a series of assembly steps where intermediates of distinct composition and architecture can be identified. In particular, atypical crescent-shaped precursors of immature particles have generated much controversy regarding their structure and the origin of their lipidic membrane. Here, we used a combination of X-ray crystallography and electron microscopy to investigate the role of a crucial structural component of viral crescents called D13. Our atomic structure of D13 firmly establishes an evolutionary link between poxviruses and a group of large DNA viruses. In addition, we show that, when tethered to artificial membranes, this protein assembles into a scaffold analogous to that in immature particles. The resulting pseudo-atomic model of the honeycomb lattice reveals similarities to the mimivirus, which suggests that giant viral shells use common assembly principles. Overall, our findings reveal that poxviruses utilize an ancestral lipid-remodeling strategy common to DNA viruses infecting all kingdoms of life. They also provide a basis for structure-based design of assembly inhibitors against poxvirus pathogens.

## Introduction

Despite the eradication of smallpox by vaccination, poxviruses remain a concern for human health because of the threat of deliberate release of the smallpox virus and occasional zoonosis [1]. This viral family contains some of the largest known viruses that require over 80 proteins for their multi-step assembly [2]. This process has been extensively characterized through studies of vaccinia virus, the prototype of the *Poxviridae* family, using electron microscopy (EM) and temperature-sensitive or conditional mutants. Assembly of vaccinia virus (Figure S1) is initiated through the formation of crescent-like precursors, followed by conversion of these precursors into spherical immature virions (IV). Next, a dramatic morphological change accompanied by proteolytic cleavage of several structural proteins produces the characteristic brick-shaped mature virions. Subsequently, the virions may acquire an additional outer membrane from trans-Golgi cisternae to form ‘wrapped’ virions and eventually exit the cell through membrane fusion to produce a third infectious form called extracellular virion [2], [3].

While the architecture of the intracellular mature virion has been recently elucidated by cryo-electron tomography [4], the structure of the crescents and immature particles poses questions on the origin and organization of their surrounding membrane. In particular, no continuity could be established between the lipid membranes of crescents and cellular compartments, which led to the early proposal of *de novo* synthesis [5]. Instead, recent studies suggest that this membrane originates from open-ended lipid sheets [6], [7]. This is consistent with evidence from deep-etch electron microscopy [8] and tomography data [6], [7] that crescents and immature particles have a single lipid bilayer rather than a flattened double membrane, thereby settling a 20-year old controversy. The crescents are covered by a protein scaffold that is primarily comprised of the D13 protein [7]. This protein has no predicted trans-membrane domain and requires the N-terminal region of the A17 integral membrane protein to form a honeycomb lattice on the external surface of crescents and IVs [9], [10]. D13 is the target of rifampicin that reversibly blocks morphogenesis before the IV stage and results in the relocation of D13 into cytoplasmic inclusion bodies [11]. Despite significant advances in the description of the architecture of crescents and IVs, the molecular mechanisms governing this step in poxvirus morphogenesis remain unclear.

Here we describe the 2.6 Å crystal structure of the vaccinia virus protein D13 and establish its membrane remodeling capacities by analysis of 2-D crystals and reconstituted IV-like particles using electron microscopy.

## Results/Discussion

### D13 is a double-barrel trimer with a head domain of novel fold

**Figure 1:**
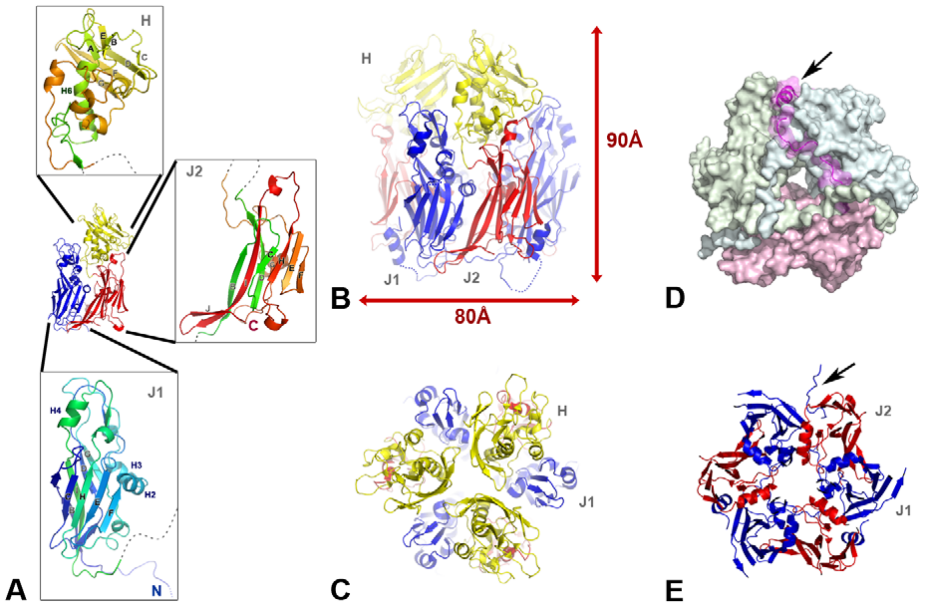
The scaffolding protein D13 of poxvirus is a trimeric double-barrel capsid protein. (**A**) The three structural domains of D13 are two successive jellyrolls (J1 and J2 colored in blue and red respectively), and a “head” domain (H colored in yellow) with a novel β-sandwich fold partially wrapped around a helix projecting away from the domain J2. Expanded details of these domains are shown as insets with a blue-to-red gradient from the N- to C-terminus of the protein. (**B**, **C**, **E**) Cartoon representation of the D13 trimer with each monomer colored as in A. The trimer is viewed in profile oriented on the viral membrane (B) and normal to the membrane as seen from the outside of the immature virion at different heights in the trimer centered on the head (C) or the J1/J2 domains (E). The arrow points to the portion of the His_6_-tag (residues −11 to 1) visible in one of the three subunits. (**D**). Domain swapping of the N-terminal loop. The molecular surface of the three subunits of a D13 trimer is represented. This surface is semi-transparent for residues 1–31 of one subunit. The cartoon representation of the N-terminal loop reveals the packing of the first helix (arrow) between the J1 and J2 domains of the subunits colored in blue and green respectively.

The crystal structure of vaccinia virus D13 reveals a double-barrel organization that consists of two jellyrolls (J1 and J2) decorated by another β-sandwich forming the head domain (Figure 1A). Both J1 and J2 domains adopt the classical jellyroll fold commonly found in viral capsids and characterized by a BIDG-CHEF β-strand order when named sequentially from B to H in the primary sequence. These two domains are structurally homologous, despite a sequence identity of only 9% between them (root mean square deviation or r.m.s.d. of 3.25 Å for 126 equivalent C_α_ atoms). In the J1 domain, the extended J1_HI_, J1_DE_ and J1_FG_ loops cluster on one side of the jellyroll forming a small sub-domain that packs against the longer J1_BIDG_ sheet of the sandwich. The head domain is inserted in the J2_DE_ loop and projects away from J2. This domain consists of a four-turn amphipathic helix followed by a β-sandwich that partially wraps around the N-terminal helix. The order of the 7 strands in the β-sandwich, named sequentially from A to G in the primary sequence is AEB-GFDC. No significant similarity to this fold was detected in the Protein Data Bank (i.e. no secondary-structure match accounted for 40% or more of the head domain).

D13 forms a trimer [9], [12] with a marked pseudo-hexagonal shape (Figure 1B–E) resulting from a parallel arrangement of J1 and J2. The pseudo-symmetry breaks down for the head domains that adopt a trefoil shape in the trimer with large gaps between each of the head domains. Each subunit buries a total of 2850 Å^2^ or 16% of its solvent accessible surface upon trimer formation. The trimeric assembly involves mostly interactions between J1 and J2 representing an interaction area of about 1265 Å^2^. The three head domains interact with each other at the center of the trimer but only through a small surface area of 170 Å^2^ (Table S1).

The trimer is hollow and presents a central channel with a diameter varying between 14 Å and 35 Å. Further stability of the trimers is derived from an N-terminal arm that forms an amphipathic helix, which extends across the pseudo-hexagonal base and packs against the opposite J1 and J2 domains of the other two subunits (Figure 1D).

### D13 is homologous to capsid proteins of icosahedral DNA viruses

**Figure 2:**
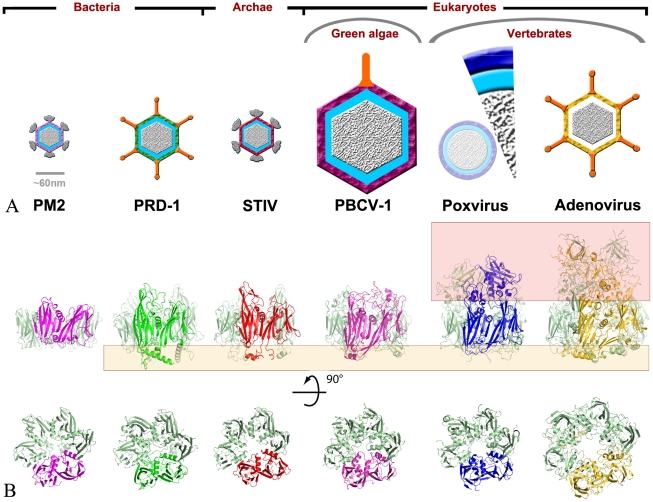
D13 is an atypical member of the double-barrel capsid family of viruses infecting all kingdoms of life. (**A**) Schematic representations of virions containing double-barrel capsid proteins, grouped according to the viral hosts. The schematics are to scale except for the immature vaccinia virion that is represented both as a 1/8^th^ slice to scale and a reduced schematic of the complete particle. Poxvirus immature particles are by far the largest objects and the only one lacking infectivity and an icosahedral symmetry. Apart from adenovirus, all viruses contain an internal lipid membrane indicated in blue. The double-barrel capsid layers are shown for PM2 (pink), PRD-1 (green), STIV (red), PBCV-1 (magenta), vaccinia virus (blue) and adenovirus (yellow). Appendages are represented in grey for turreted viruses and orange for viral fibers. (**B**) Cartoon representations of the double-barrel capsid proteins characterized by the highly-conserved central double-barrel domain with the same coloring scheme for one of the subunit as in (A). The bottom panels represent the proteins viewed from the outside of the particle and the top panels include those for an orthogonal view. In contrast, when present, the “head” and “feet” regions highlighted in red and orange boxes respectively correspond to the most dissimilar domains amongst the capsid proteins.

Structural comparison reveals close similarity between D13 and capsid proteins of several large DNA viruses (Figure 2 and Table S2). The most similar protein is the major capsid protein VP54 of PBCV-1 [13], a virus of the *Phycodnaviridae* family that infects unicellular green algae. PBCV-1 belongs to the Nucleo-Cytoplasmic Large DNA Viruses (NCLDV) lineage that includes the poxviruses, phycodnaviruses, asfaviruses, iridoviruses and mimivirus. The monophyletic origin of these distantly-related viruses was established by advanced sequence analysis that identified a core set of 41 proteins conserved in all these viruses [14]. However, poxviruses appeared to form a separate clade in this classification in part because J2 was predicted to adopt a fold structurally unrelated to the jellyroll domain found in all other double-barrel capsid proteins. Instead, we found that the similarity between D13 and VP54 extends over the two jellyrolls with an r.m.s.d. of 2.4 Å for 80% of the residues in J1 and J2 (Table S2). This finding supports the notion that the two jellyrolls result from a duplication event that predates the divergence of the clades currently observed for all NCLDV members.

In addition to the homology with the NCLDV lineage of eukaryotic viruses, the structure of D13 harbors strong similarity with the double-barrel protein family including the major capsid proteins of PRD1 [15], PM2 [16] and STIV [17]. These viruses are DNA viruses infecting Gram-negative bacteria, *Pseudoalteromonas* bacteria and archae respectively (Figure 2) and, in contrast to the NCLDV, they are too distant to be linked to poxviruses by sequence analysis. Despite having prokaryotic hosts and icosahedral capsids, these viruses contain double-barrel proteins that share not only the same topology and trimeric organization as D13 but also an analogous function as the major component of the protein shell enclosing the viral membrane.

The adenovirus hexon protein is also considered to be a member of the double-barrel capsid family based on structural comparison with the major capsid protein of PRD1 [15]. Initially, we did not detect significant structural similarity with D13 based on a simple search for secondary-structure matches (Table S2). However, a careful analysis reveals that the double-barrel cores of D13 and adenovirus hexon share very similar topologies (Figure S2) and adopt analogous quaternary arrangements within the trimer and on the viral surface (Figure 2). In addition to the double-barrel base, the hexon also has a head domain forming a trimeric spike analogous to that observed in D13. However, the hexon head domain is composed of elaborate loops contributed by both jellyrolls rather than being inserted in the J2_DE_ loop and is thus unrelated in structure to the head domain of D13 (Figure 2 and S2). Given this convoluted structure characterized by a complex head domain and the absence of an internal membrane in the adenovirus capsid [18], the hexon constitutes a more distant member of this protein family than D13.

The unambiguous structural homology between D13 and the double-barrel proteins defines a minimal core of these capsid proteins. This core extends beyond the basic jellyroll sandwiches through the inclusion of the conserved helices inserted in loops J1_FG_ and J2_FG_ (Figure S2). These helices constitute a constriction in the central channel of the D13 trimer. On the other hand, the bases and the spikes of the trimers are highly variable in their size and structure (Figure 2), which may reflect virus-specific interactions with inner layers of the virion and different functions for the head domains in the life cycle of each virus.

### Do poxviruses belong to the PRD1-adeno lineage?

Homology between the proteins of the double barrel family has been proposed to support phylogenetic relationships between the respective viruses, which was used as a signature for the so-called PRD1-adeno lineage of viruses [19], [20]. The place of poxviruses with regard to the PRD1-adeno lineage remained uncertain in part because of their distinctive brick-shaped morphology that lacks obvious icosahedral symmetry and sets them apart from all other DNA viruses. Even when poxviruses were linked to NCLDVs by sequence analysis, they remained segregated in a highly divergent clade [14].

The unambiguous link uncovered here between the D13 protein and the double barrel capsid family lends support to the inclusion of poxviruses as *bona fide* members of the PRD1-adeno lineage. In this scenario, poxviruses would have evolved from a precursor of the PRD1-adeno lineage that would have subsequently lost its infectiousness and icosahedral structure when the more complex brick-shaped infectious particles evolved. However, given the remarkable ability of poxviruses to hijack viral and cellular mechanisms for their own replication, many other scenarios are possible for instance involving horizontal transfer of the D13 protein. Further structure-function analyses of poxvirus core components will be needed to provide convergent evidence establishing the relationship of poxviruses to the PRD1-adeno lineage.

### *In vitro* self-assembly of D13

**Figure 3:**
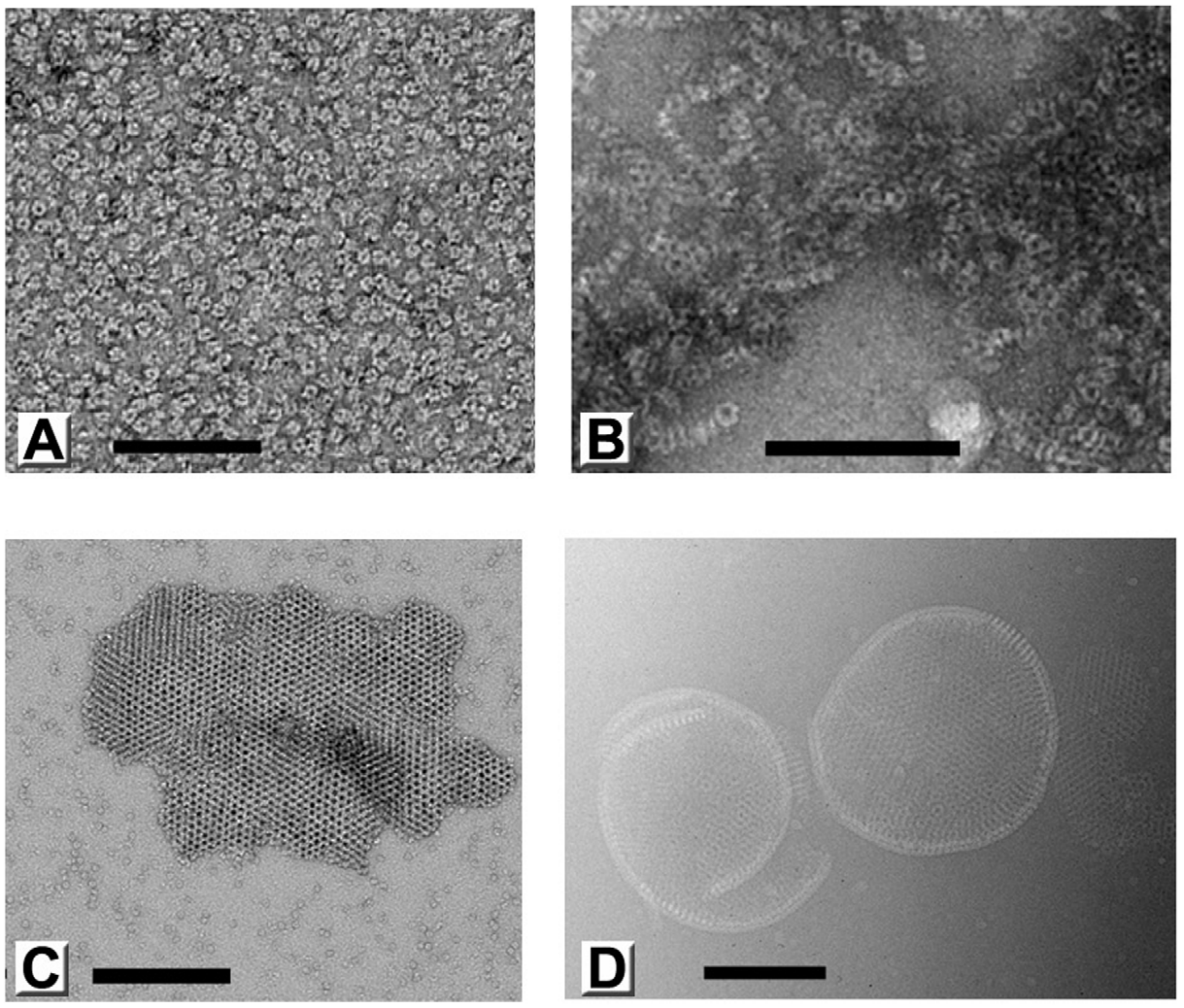
Polymorphic assemblies of purified D13 visualized by electron microscopy. (**A**) An electron micrograph of homogeneous purified D13 trimers visualized by uranyl acetate staining (negative staining). (**B**) Self-assembly of short tubular objects is observed in the absence of arginine and glutamate additives and when the His_6_-tag is removed by proteolytic digest. When purified protein is dialyzed into low ionic strength buffers, assembly is observed in the form of small crystalline patches (negatively stained) (**C**) and irregular spherical assemblies as observed by cryoEM (**D**). Scale bars represent 100 nm (A) and 200 nm (B–D).

Like many capsid proteins [21], purified D13 has an intrinsic propensity to self-interact and to assemble into polymorphic objects such as trimers, tubular assemblies and large aggregates (Figure 3). In our hands, self-assembly of D13 was particularly promoted by low ionic strength while the addition of 50 mM arginine/glutamate maintained the protein in a monodisperse, trimeric state. Dialysis into a low-salt buffer resulted in the frequent formation of small 2-D crystalline patches (Figure 3C). These objects are reminiscent of 2-D crystals observed *in vitro* for the orthologue ORFV075 of orf virus [12], those identified for D13^D513G^ in transfected cells [9], and possibly similar to the smaller objects observed in infected cells by Chlanda and coll. [7].

In addition, we observed large assembly products presenting a curved honeycomb lattice, which have not been described before (Figure 3D). The honeycomb lattice was obviously bereft of any underlying lipid membrane and displayed continuous curvature consistent with an incomplete spherical morphology when suspended in vitreous ice. The objects formed were very heterogeneous in size and shape, but always displayed a latticed morphology on their surface. We observed mostly curved sheets and only one fully closed particle (1.5% of all objects). The thickness of these sheets is 96 Å±5 Å (standard deviation; sd) and the trimer-to-trimer distance on the sheets is 75 Å±2 Å (sd). These geometrical parameters are comparable to the corresponding features in immature particles [8].

### D13 assembly on lipid membranes

**Figure 4 ppat-1002239-g004:**
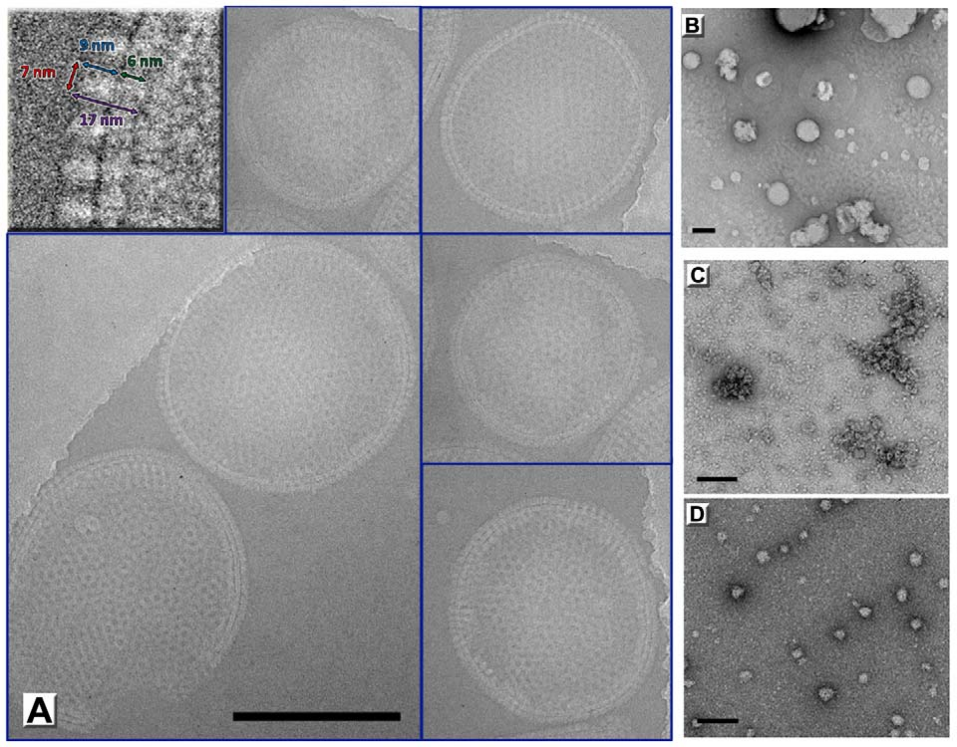
Immature virion-like particles formed by D13 on artificial membranes. (**A**) D13 was incubated with a mixture of dissolved lipids (59% DOPC, 18% DOPE, 3% DOPS doped with 20% of nickel loaded DOGS-NTA) and detergent (4% n-βOG). After dialyzing away the detergent, assembly of large spherical shells is observed by cryoEM only when His_6_-tagged D13 is present. The top left inset shows a blown up area where the D13 spikes are readily visible. (B–D) Electron micrographs of negatively stained samples of control experiments using no protein (**B**), D13 after His_6_-tag removal by rTEV protease digest (**C**), and a non-relevant His_6_-tagged protein (**D**). Scale bars represent 200 nm.

To further gain insight into the assembly of immature virions, we established two different *in vitro* assembly systems on lipid surfaces that exploit the ability of His_6_-D13 to bind nickel-chelating lipids. In the first system, we formed artificial liposomes using detergent-solubilized lipids with a composition close to that of the endoplasmic reticulum but doped with nickel-chelating lipids. These liposomes are intrinsically heterogeneous in size and shape (Figure 4B). However when purified D13 trimer was added during the liposome formation, large spherical objects were produced (Figure 4A). Contrary to the self-assembly observed for isolated D13, the formation of these objects appeared to be independent of the buffer molarity and ionic strength. Importantly, the induced lipid remodeling is specific for the His_6_-D13 lipid interaction since, these objects were not observed with untagged D13 or a His_6_-tagged protein not relevant to poxvirus biology (Figure 4C,D). When suspended in vitrified buffer, 34% of the assemblies were closed particles, either spherical (19 out of 25) or somewhat distorted (6 out of 25). The remaining assemblies also adopted a curved lattice but were open-ended, indicating that they failed to close or were disrupted during sample preparation. All these objects clearly displayed a continuous, unilamellar lipid membrane underneath the D13 scaffold (Figure 4A). Under the given assembly condition no irregular liposomes or membranous structures free of D13 were observed. The size of closed particles is relatively heterogeneous, ranging from 150 nm to 350 nm in diameter and, as in the immature virions, lack global icosahedral symmetry. The thickness of the protein scaffolding including the underlying lipid layer is 170 Å±7 Å (sd) including a protein spike 92 Å±7 Å (sd) in height. The inter-trimer distances are 75 Å±2 Å (sd) (Figure 4A). The organization of the spikes on the surface of these particles is in good agreement with the spacing in the D13 honeycomb-shaped lattice observed *in vivo* on the immature virions and crescents [7], [8]. Overall, these assemblies mimic the immature particles remarkably well given that they are only composed of D13 and lipids without any other viral and/or cellular components of immature virions. The absence of these multiple components of immature virions may account for the large size distribution and the lower efficiency to form fully closed particles that are observed in the *in vitro* assembly system.

**Figure 5:**
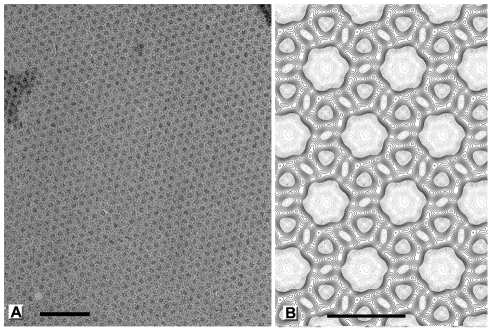
Honeycomb lattice formed by D13 on artificial membranes. (**A**) When a lipid monolayer composed of egg PC and PG doped with nickel loaded DOGS-NTA was carefully formed to produce a flat membrane at the air-liquid interface, D13 trimers formed 2-D crystals. These crystals are relatively large and undistorted areas could be identified by optical diffraction. (**B**) Projection map computed from the negative stain images of the 2-D crystals without imposing any symmetry. Similarity is evident with the honeycomb lattices observed *in vivo* on the surface of immature particles (Figure 7 of reference [8]) and on flat sheets of the D13^D513G^ protein (Figure 4 of reference [9]). Scale bars represent 100 nm in panel (A) and 14 nm in panels (B).

The heterogeneity and absence of global symmetry of the IV-like assemblies precluded higher resolution analysis. However, regular 2-D crystals of D13 were produced on a lipid monolayer doped with nickel-chelating lipids (Figure 5A). 2-D crystals were readily obtained but seldom the lattice was continuous over more than 0.5 µm suggesting that interactions mediating the 2-D crystallization also have the propensity to induce curvature. Such deformations were also observed for orfv075 2-D crystals formed on lipid monolayers [12].

**Figure 6:**
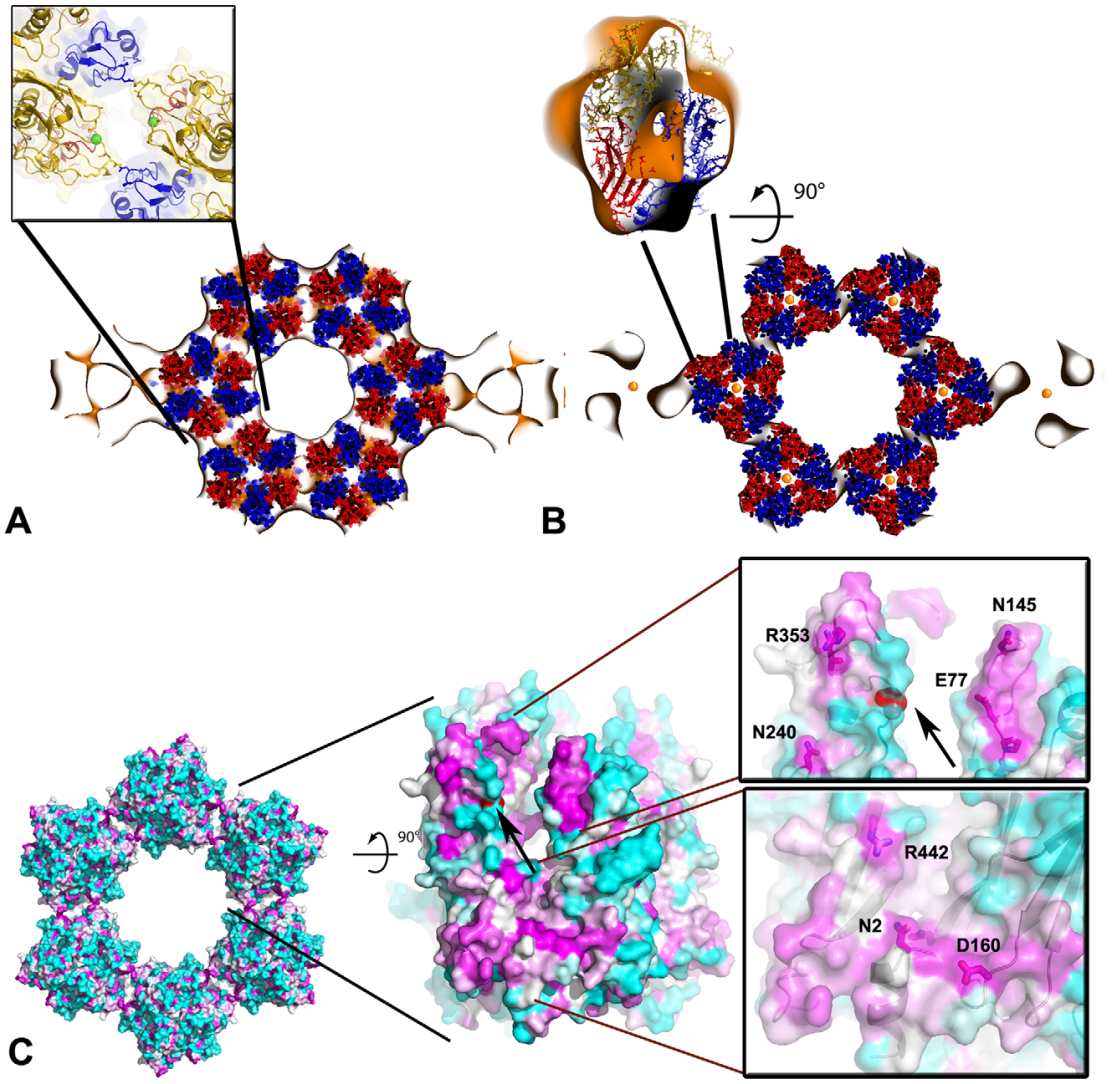
Pseudo-atomic model of the honeycombed assembly of D13. Docking of the atomic structure of D13 trimer into the ∼20 Å resolution 3-D density map generated by electron crystallography of 2-D crystals shown in Figure 5A. (**A**) View of a slab approximately through the middle of the trimers showing the p6 honeycombed assembly of D13 looking onto the membrane plane from ‘outside’. The inset represents a detailed view of the interface between two D13 trimers involving J1-J2 contacts. Residue G_513_ is shown as a sphere colored in green. (**B**) Slab proximal to the lipid membrane indicating differences between the X-ray crystal structure and the EM structure. The inset shows an orthogonal view of the fit of the atomic model of a D13 trimer colored as in Figure 1 into an excised volume of density from the EM 3-D map. (**C**) Conserved domains in D13 mapped onto the molecular surface. The surface is colored in a cyan-white-magenta gradient from the least to the most conserved residues as estimated from an alignment of 16 unique sequences of poxviruses. Residue G_513_ is highlighted in red (arrow). The two panels represent detailed views of the most highly conserved regions of D13. Side chains of strictly conserved residues are indicated as sticks within the semi-transparent surface and residues close to lattice contacts are labeled.

The ∼20 Å structure generated using images of tilted 2-D crystals preserved in uranyl acetate stain reveals striking similarities between the lattice of Ni-lipid tethered His_6_-tagged D13 and the honeycomb scaffold observed *in vivo* (Figures 5B and S3). These lattices share a local p6 plane group of symmetry resulting in a honeycomb morphology with inter-trimer distances of ∼80 Å. The lattice parameters of the 2-D crystals are a = b = 138.5 Å, which is in good agreement with that for the native D13 scaffold [8]. Moreover, the trimeric building block observed in the 2-D crystal is in good agreement with the high-resolution crystal structure. Thus, the large, tripod-shaped base domain at the membrane interface corresponds to the J1 and J2 domains while the radial projection is occupied by the head domain with its characteristic trefoil shape (Figure 6A–B). Overall, the very similar molecular organization strongly suggests that the interactions observed in the 2-D crystals are relevant to the assembly of immature virions.

### Molecular model of the honeycomb lattice

To provide a molecular model of the 2-D crystal lattice, the crystal structure of D13 was fitted into the electron microscopy-derived 3-D density map (Figure 6A,B). The fit is constrained both by the lattice symmetry and the prominent trefoil shape of the head domain (real space correlation of 73.5%). Minor structural differences are observed at the top of the head domain but the main area of disparity between the two structures is located at the membrane proximal region. The N-terminal arm is situated at the base of D13 and, in the X-ray structure it folds back onto the J1–J2 domain projecting towards the head domain (Figure 1D–E). This arm contains the His_6_-tag and appears to have been “pulled” towards the membrane in the 2-D crystal to mediate interaction with lipid-bound nickel molecules. Thus, we attributed the additional density seen in the EM structure to the N-terminal arm of D13 (Figure 6B).

The 3-D model of the honeycomb lattice reveals a loosely connected network of trimers that interact at three of their pseudo-hexagonal faces while the three other faces are oriented towards the large channel at the center of each ring of six trimers. Trimers interact primarily via the protruding loop J1_FG_ and loop EF of the head domain. The base of the trimer and loop J1_DE_ are also in close proximity and may also contribute to the assembly of the scaffold *in vivo* (Figure 6A and S4). The contact area is limited in size, which is uncharacteristic of a viral capsid [22], although the loops involved may rearrange to form tighter interactions in immature particles.

Molecular surfaces that are most conserved in sequence across the *Poxviridae* family are located at the inter-trimer contact area while variable regions face the large channels in the honeycomb lattice (Figure 6C). The conserved surfaces create two grooves spanning almost the entire height of the trimer. The first groove is at the base of the trimer while the second is located between the J2 and head domains ∼50 Å above the membrane surface. This latter region contains the residue D_513_ (Figure 6C and S4) whose mutation to a glycine abolishes interaction of D13 with lipid membranes without affecting its ability to self-assemble *in vivo* [9]. We determined the crystal structure of both native D13 and D13^D513G^. The two models only differ in minor loop movements although several loops could not be modeled in the native D13 structure because of its lower resolution (Figure S5). The two structures have an r.m.s.d. of 0.5 Å for all equivalent atoms. In our model, residue D_513_ is ∼14 Å away from the adjacent trimer. The 3-D density map generated by electron microscopy also reveals a large gap between residue D_513_ and the closest surface of the neighboring trimer confirming that D_513_ is apparently not mediating a direct inter-trimer interaction in the 2-D lattice. Thus, mutation of residue D_513_ does not appear to directly destabilize the fold of D13 or the honeycomb scaffold. In fact, D13^D513G^ appears to self-assemble more readily than the native protein forming large sheets *in vivo* [9]. One plausible explanation for the assembly defect observed with the D13^D513G^ protein is that the mutation indirectly induces premature or distorted self-assembly of D13, sequestering the protein away from the poxvirus assembly site. The surface-exposed residue D_513_, which is in a conserved patch of D13 is available for interaction with a potential cellular or viral partner. Thus, mutation of this residue could also disrupt an interaction essential for the docking of D13 onto the crescent-shaped membranes.

**Figure 7:**
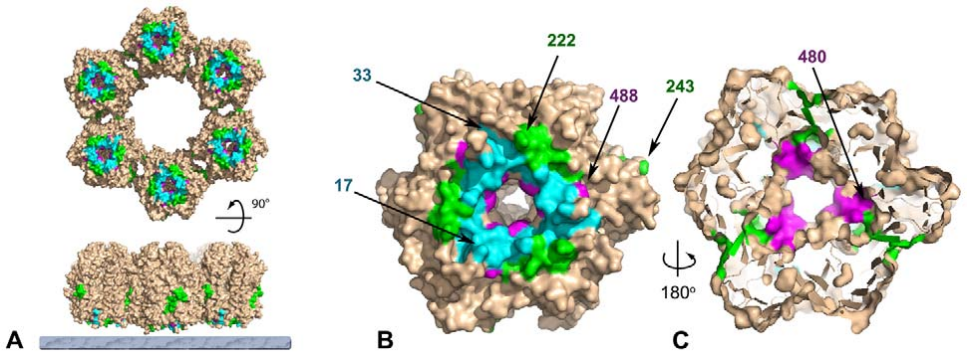
Mutations in response to rifampicin cluster at the base of the D13 trimer. Surface rendered view of a D13 trimer indicating the three sites, defined by Charity and coll. [23], where mutations that produce resistance to the antiviral effect of rifampicin cluster. Site I (residues 17–33; cyan), II (residues 222–243; magenta) and III (residues 480–488; green) are highlighted with the rest of the molecular surface colored brown. The first and last residues of each cluster are indicated in (B) and (C) for one of the subunit. Panel (**A**) represents a hexameric ring of D13 trimers on the viral membrane (bottom, grey layer) and normal to the membrane viewed from the inside of the particle (top). Panel (**B**) represents a view from inside the particle through the membrane similar to those in (A) and (**C**) and represents a slice approximately through the centre of the double-barrel domain as viewed from the outside of the particle.

Mutations in D13 that generate rifampicin-resistant viruses [23] cluster in three contiguous regions that all map to the same membrane proximal region of D13 and cover about half of the base, near the central channel of the trimer (Figure 7). One of these clusters is located in the channel itself with restricted solvent access. Overall, this region is too large to represent a single site of binding for the rifampicin molecule (823Da). Soaks and co-crystallization experiments with rifampicin have so far failed to reveal a complex with D13. In view of this, it is unclear whether these mutations destabilize direct binding of rifampicin to D13 or alter the interaction of D13 with a viral partner rendering it insensitive to the effect of rifampicin.

### Assembly of the D13 scaffold

**Figure 8:**
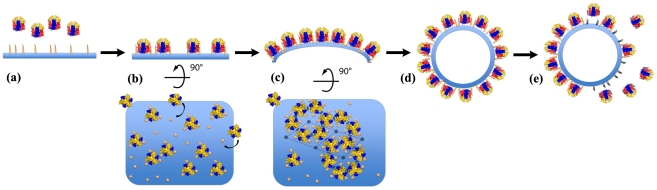
Model of the immature virion-like particle assembly. The color scheme used for D13 trimers is the same as in Figure 1. The membrane is represented in blue and the nickel-lipids are represented as yellow crosses and spikes. (**a**) Attachment sites for the His_6_-tag promote membrane association of D13 *in vitro*. This interaction appears to act as a surrogate of viral binding partner(s) embedded in the membrane such as the A17 protein. (**b**) Subsequently, D13 trimers dock onto the surface and form a honeycomb lattice. (**c**) The apparently continuous curvature of the honeycomb lattice results in a crescent-like formation. (**d**) This structure is eventually extended to form a spherical particle. Defects in the lattice are not depicted here but they are necessary to achieve a closed shell and were frequently observed on immature virions in previous studies [8]. (**e**) Because D13 trimers do not interact directly with the membrane, cleavage of the attachment anchor, such as the N-terminal region of A17, may be sufficient to induce the detachment of either single trimers or assembled D13 sheets from the enveloped particle.

A model for the assembly of immature virion assembly is presented in Figure 8. Based on our *in vitro* assembly data, docking of the D13 trimer onto a lipidic support promotes large-scale, regular assembly of D13 rather than irregular objects (Figure 8a). Similarly, when interaction with membranes is altered *in vivo* either by rifampicin [24] or by the D_513_G mutation, D13 relocates away from the assembly site [9]. *In vitro*, docking on this membrane support appears to be sufficient to generate a honeycomb lattice (Figure 8b), which involves relatively limited interactions (Figure 6 and S4). In turn, this lattice alone, analogous to the scaffold observed *in vivo* on crescents, is able to remodel the underlying membrane inducing a curvature compatible with closed particles of approximately 300 nm in diameter (Figure 8c,d). Three-dimensional information, such as cryo-tomography of the immature virion-like particles, will be required to analyze in detail the specific inter-trimer contacts and interaction with membrane-bound partner(s) that induce membrane curvature. *In vivo*, the growth of crescents elicits the appearance of defects necessary to form a closed shell and identified as pentamers and heptamers by Heuser and coll. [8]. We did not observe contacts compatible with pentameric assembly in the two forms of 3-D crystals (Figure S5) or in any of the *in vitro* assemblies (Figures 3–5). This suggests that these defects may be relatively rare *in vitro* and a result of the induced curvature rather than a driving force. However, *in vivo*, a higher efficiency of closure of the particles and an increased homogeneity in size are observed and could result from the contribution of other proteins of the immature particle inducing pentameric or heptameric assemblies in the scaffold.

A recent study established that A17 represents at least part of the membrane docking signal for D13 and that cleavage of the N-terminus of A17 by the I7 viral protease correlates with the disassembly of the scaffold [10]. Because D13 does not interact with the membrane directly (Figure 4C) and the lattice interactions are relatively limited (Figure 6 and S4), our model of the scaffold is compatible with such a dismantling mechanism induced by removal of the membrane-docking signal (Figure 8e).

### Comparison with the giant mimivirus capsid

**Figure 9:**
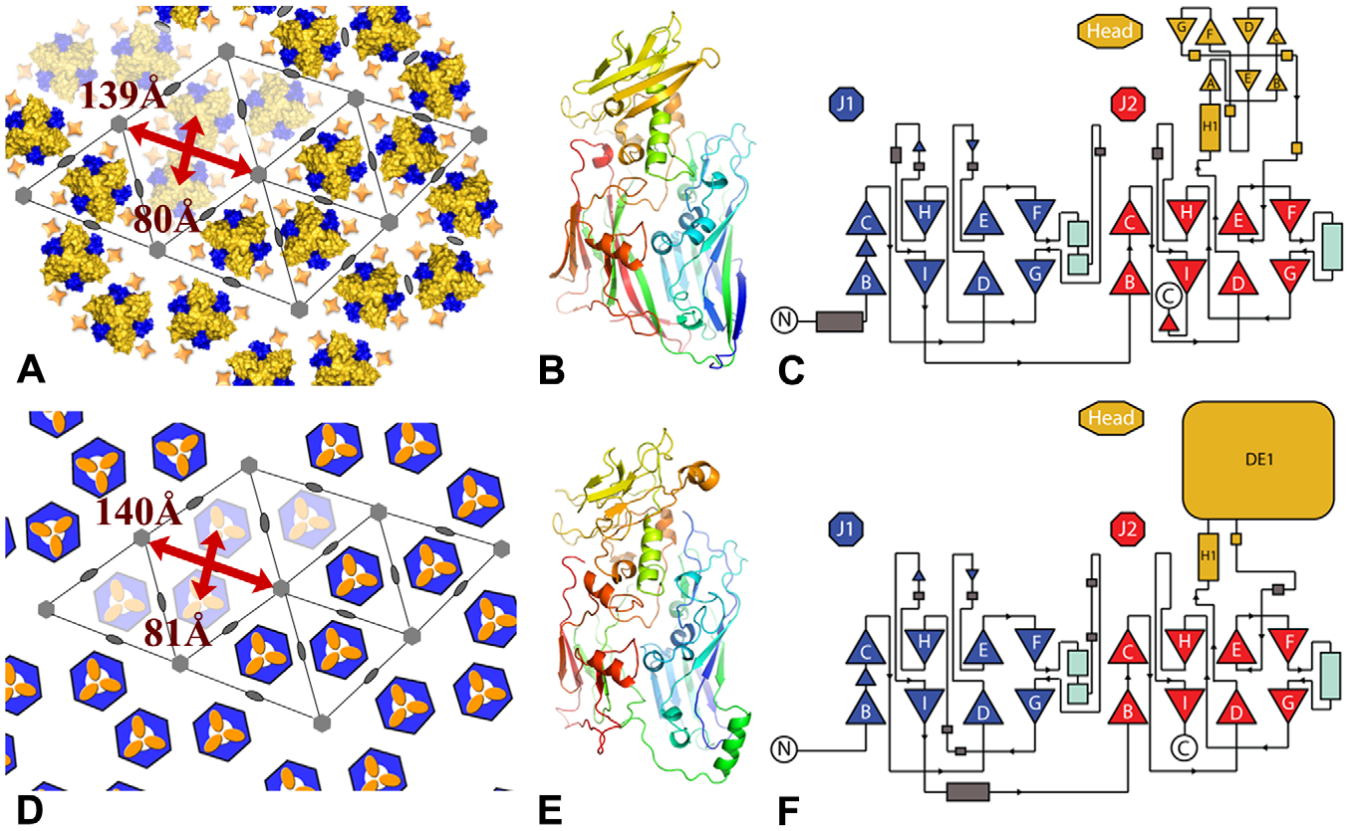
Comparison between the lattices and capsids of vaccinia virus and mimivirus. (**A**, **D**) Honeycomb lattices formed by vaccinia virus D13 and mimivirus P1 respectively. The color scheme is the same as in Figure 5 and the mimivirus capsomer is schematized by a hexagonal base and a head domain colored in blue and yellow respectively [25]. The p6 symmetry is only local and other members of the double-barrel lineage adopt a local p3 lattice with an additional trimer instead of a gap at the 6-fold axis. The positions of the 2- and 6-fold symmetry axes are indicated by ellipses and hexagons. Lattice parameters are indicated in red. (**B**, **E**) Analogous views of D13 and a model of the mimivirus P1 capsid protein determined by the Phyre2 server with a blue-red gradient from N- to C-terminus. The confidence is 99.83% with 457 aligned residues sharing 21% sequence identity. The head domain of mimivirus is predicted to be inserted in the J2_DE_ loop like its counterpart in D13 and to share a similar spatial arrangement at the top of the spike. (**C**, **F**) Topology diagrams for D13 and P1. J1 and J2 are represented in blue and red respectively, the head domains are shown in yellow. The head domain of P1 is only represented schematically as a box.

The honeycomb arrangement of D13 described here is remarkably similar to the flat sheets observed on the surface of the mimivirus [25]: both lattices consist of a loosely packed honeycomb lattice with an apparent p6 plane group of symmetry (a = b≈140 Å) that is characterized by large openings at the center of the hexagonal cell (Figure 9A,D). In addition, our homology modeling of the mimivirus structure predicts not only very similar J1 and J2 domains as in D13 but also a head domain of similar size and location in the 3-D structure (i.e. 190 residues inserted in the J2_DE_ loop; Figure 9). Thus the double-barrel proteins of poxvirus and mimivirus may be more closely related than assumed before. The similar structural components of these giant viruses clearly depart from those of smaller icosahedral viruses of the double-barrel lineage that all adopt a compact local p3 symmetry (a = b≈75 Å). Future structural studies will reveal whether the apparent similarities in the architectures of poxviruses and mimivirus reflect homologous molecular mechanisms to assemble what are the largest known viral capsids.

### Concluding remarks

Our findings reveal that the first morphogenesis step of poxviruses rely on ancestral lipid-remodeling strategies found in a lineage of icosahedral DNA viruses. The scaffolding protein D13 constitutes a driving force in this process as it has the intrinsic ability to form immature-virion like particles in the absence of any other viral factors. Indeed, neutralization of D13 either by point mutation or rifampicin completely inhibits viral assembly. The unique role of D13 in assembly contrasts with the redundancy otherwise observed in poxvirus-host interactions, such as the multiple anti-apoptotic strategies. The formation of immature particles may thus represent the Achilles' heel of poxvirus thereby validating D13 as a target for the development of antivirals against poxvirus pathogens.

## Methods

### Expression and purification of D13

DNA coding for D13 was amplified by PCR from genomic material of Western Reserve strain of vaccinia virus (kindly provided by Prof. A.A. Mercer) and cloned into the pPROEX-Hta vector (Invitrogen) using sequence- and ligation-independent cloning (InFusion) because the D13 gene contained most common restriction sites. The D13 gene was inserted in frame with a 5′ His_6_-tag and a Tobacco Etch Virus (rTEV) protease recognition site. Cells were grown at 37°C to OD_600_ = 0.6. Cells were left to cool at room temperature, induced with 0.25 mM IPTG and incubated overnight at 20°C. Cells were lysed by sonication in 30 ml lysis buffer containing 50 mM Tris-HCl pH 8, 300 mM NaCl, 10% glycerol, 5 mg lysozyme, 2 mM β-mercaptoethanol and benzonase nuclease. The soluble fraction was isolated by centrifugation at 4000× g for 30 min. The filtered soluble fraction of His_6_-tagged D13 was purified by immobilized metal affinity chromatography (IMAC) using a 50–500 mM gradient of imidazole over 50 mL and dialyzed against 2 L of storage buffer containing 50 mM Tris/HCl (pH 8.0), 500 mM NaCl and 50 mM L-arginine and 50 mM L-glutamic acid and 2 mM β-mercaptoethanol to prevent aggregation. When required, His_6_-tag was cleaved during overnight dialysis at 4°C by adding rTEV protease to D13 at approximately 1∶10 (w∶w) ratio. Further purification was carried out by size-exclusion chromatography (SEC) using Superdex HR 200 16/60 column (GE Healthcare), equilibrated with the storage buffer, on Äkta Purifier (GE Healthcare) operating at an ambient temperature (20–25°C). The fractions collected from SEC were analyzed by SDS-PAGE.

### Mutagenesis

The D_513_G mutation was introduced in the D13 construct by site-directed mutagenesis. Mutation was introduced by PCR using Phusion High-Fidelity DNA Polymerase (Finnzymes) and primers

5′–GTTTATTCCACCATGGGTGTCAACCATCCAATC (forward);

5′-GATTGGATGGTTGACACCCATGGTGGAATAAAC (reverse).

DpnI was added to the PCR product and incubated at 37°C for 1 hour. The reaction mix was directly used to transform chemically-competent DH5α cells. We used the same purification procedures for D13^D513G^ than for the native protein.

### D13 crystallization

The native D13 and D13^D513G^ proteins were concentrated to 8 mg.mL^−1^ and 2.9 mg.mL^−1^ respectively without cleaving the His_6_-tag. Concentrated protein was subjected to crystallization trials on a Rigaku CrystalMation robotic system in Intelliplate 96-well crystallization plates. The reservoir volume is 50 µL and sitting drops contain 100 nL of protein solution and 100 nL of precipitant. Initial hits were optimized using the hanging drop method with 2 µL drops and 500 µL precipitant in each well. Large but thin hexagonal plaques (∼200 µm ×200 µm ×10 µm) were obtained for the D13 protein with the best crystals produced in 200 mM NaBr, 20% PEG 3550 and Tris pH 8.0. The D13^D513G^ protein produced hexagonal bipyramidal and spindle-shaped crystals in several conditions containing carboxylic acids as precipitant (e.g. acetate, formate, malate). The best crystals measured approximately 200 µm in diameter and were produced in 2.3 M Na formate, 100 mM Bis-Tris pH 6.5.

### Data collection and structure determination

Crystals were transferred in a cryopotectant solution containing 200 mM NaBr, 24% PEG 3350, Tris 100 mM pH 8.0 and 20% ethylene glycol for D13 and 2.6 M Na formate, 100 mM MES pH 5.8, 30% glycerol for D13^D513G^ and flash frozen in liquid nitrogen. Data were collected at a temperature of 100K and energy of 13000keV at the X06SA of the Swiss Light Source and the MX1/MX2 beamlines of the Australian Synchrotron. For phase determination, crystals of D13^D513G^ were soaked for 1 h in 3 µL of 2.6 M Na formate, 100 mM MES pH 6.0 plus 10% of a saturated solution of either ethyl mercury chloride or KPtCl_4_. Soaked crystals were subjected to diffraction experiments as described above.

Diffraction images were processed with the XDS software (D13^D513G^) [26] or HKL2000 (native D13) [27] and the CCP4 suite [28]. The native D13 crystals exhibited a very strong anisotropy, which was particularly pronounced in the plane of the plaque. These crystals belong to the R32 space group with cell parameters of a = b = 125.126 Å, c = 370.826 Å and α = β = 90°, γ = 120° and best resolution of 3.4 Å. The D13^D513G^ crystals belong to the P6_1_22 space group with cell parameters of a = b = 189.630 Å, c = 255.130 Å and α = β = 90°, γ = 120°. They diffracted more consistently with no significant anisotropy and a resolution of 2.55 Å for non-soaked crystals, 3.0 Å and 3.1 Å for ethyl mercury chloride and KPtCl_4_ soaks respectively (Table 1). Heavy atom sites were identified by SHELX [29] and refined with AutoSHARP [30]. Phases were of sufficiently good quality to produce a readily interpretable map (Table 1) and ARP/wARP [31] produced a model containing 1550 residues in 4 chains for the 3 molecules in the asymmetric unit. The model was completed using Coot [32] and refined with Buster [33] using individual B factors and automatic NCS restraints. The final model of the D13^D513G^ trimer contains residues 15 to 547 and the last 13 residues of the His_6_-tag (-11-1) in chain A and C; 1 to 547 for chain B and the last two residues for the tag in chain B. The N- and C-termini and one loop (residues 46–48) were disordered and could not be confidently modeled. The geometry is very good as assessed by the MolProbity server [34] (1.88 Molprobity score, 6.82 clash score; both scores are in the 98^th^ percentile of best structures at 2.55 Å+/−0.25 Å). 96.6% of residues were in the favored region of the Ramachandran plot after refinement with no outliers. The final reliability factors are satisfactory with R = 17.23% and R_free_ = 19.83%.

**Table 1:** Data collection, phasing and refinement statistics.

	D13^D513G^	EtHgCl	K_2_PtCl_4_	Native D13
**Data collection**				
Space group	P6_1_22	P6_1_22	P6_1_22	R32
Cell dimensions				
*a*, *b*, *c* (Å)	189.63, 189.63, 255.13	190.06, 190.06, 258.20	187.35, 187.35, 253.87	125.13, 125.13, 370.83
*α, β, γ* (°)	90, 90, 120	90, 90, 120	90, 90, 120	90, 90, 120
Resolution (Å)	20–2.55 (2.62–2.55)*	20–3.0 (3.08–3.00)	20–3.1 (3.18–3.1)	20–3.5 (3.62–3.50)
*R*_sym_ (%)	8.0 (101.7)	14.2 (107.5)	18.6 (113.2)	15.6 (55.7)
*I*/σ*I*	26.25 (2.25)	14.09 (2.01)	18.23 (3.46)	10.4 (2.2)
Completeness (%)	99.6 (99.9)	99.3 (99.8)	99.4 (99.9)	97.9 (98.8)
Multiplicity	10.9 (9.4)	7.8 (7.8)	21.9 (22.5)	6.8 (6.4)
**Phasing** #				
Isomorphous Phasing power (centric/acentric)	-	0.63/0.58	0.34/0.32	-
Anomalous Phasing power	-	0.519	0.134	-
FOM	0.32/0.28			-
**Refinement**				
Resolution (Å)	18–2.55 (2.62–2.55)			30–3.5 (3.79–3.51)
No. reflections	87867 (6417)			13943 (2769)
*R*_work/_ *R*_free_ (%)	17.32/20.01 (22.96/27.35)			24.52/25.65 (25.01/28.26)
No. atoms				
Protein	12873			7428
Ligand (Formate)	30			-
Water	713			-
B-factors (Å^2^)				
Protein	50.1 (chain A) 58.2 (chain B) 70.2 (chain C)			102.7 (chain A) 76.7 (chain B)
Ligand/ion	70.2			-
Water	53.8			-
R.m.s deviations				
Bond lengths (Å)	0.010			0.009
Bond angles (°)	1.19			1.09

*Highest resolution shell is shown in parenthesis.

# Phasing statistics are reported for the resolution range 20–3.0 Å.

The structure of native D13 was determined by molecular replacement using Phaser implemented in Phenix [35]. Two molecules are present in the asymmetric unit and the structure was refined to 3.5 Å using Buster with one TLS group per chain (2 chains in the asymmetric unit), no B factor refinement due to the lack of resolution and automatic NCS restraints. The final model has a good geometry as assessed by the MolProbity server (Molprobity score of 2.73 in the 97^th^ percentile of 37 structures above 3.0 Å; clash score of 9.96 in the 95^th^ percentile). 93% of residues were in the favored region of the Ramachandran plot after refinement with 31 (chain A and B) and 170 (chain A) as outliers. The final reliability factors are satisfactory at this resolution with R/R_free_ of 22.3% and 27.1%.

### Assembly of D13 *in vitro*

A volume of 50 µl of the protein in the storage buffer at 0.5 mg/ml was loaded into a homemade dialysis button, sealed with a 14 kDa molecular mass cut-off membrane. The dialysis was carried out for 15–18 hours against 200 ml 10 mM HEPES (pH 7.0), 50 mM NaCl and 2 mM β-mercaptoethanol, at 4°C, promoted self-assembly. After buffer exchange, the resulting samples were examined by TEM to assess protein assembly.

The assembly of D13 in complex with lipid was carried out as follows. A mixture of lipids composed of 59% 1,2-Dioleoyl-sn-Glycero-3-Phosphocholine (DOPC), 18% 1,2-Dioleoyl-sn-Glycero-3-Phosphoethanolamine (DOPE), 3% 1,2-Dioleoyl-sn-Glycero-3-[Phospho-L-Serine] (Sodium salt) (DOPS), mimicking the lipid-composition the endoplasmic reticulum [36], doped with 20% {1,2,-dioleoyl-sn-glycero-3-[(N-(5-amino-1-carboxypntyl) iminodiacetic acid) succinyl]}-nitrilotriacetic acid-Ni^2+^ (DOGS-NTA-Ni) was used. The lipid-dissolving organic solvent was completely dried under gentle flow of nitrogen and then the lipid mixture was solubilised by adding the protein storage buffer containing 4% (w/v) n-octyl-β-D-glucoside. The His_6_-tagged protein was added to the lipid solution at 1∶2 (w∶w) lipid-to-protein ratio. The mixture was incubated under gentle stirring for 15 hours at room temperature to allow the formation of lipid-protein-detergent complex. Then the lipid-protein-detergent mixture was dialyzed extensively against the protein storage buffer at an ambient temperature (20-25°C), using a 14 kDa cut-off dialysis membrane, until complete removal of the detergent was achieved.

### 2-D crystallization of D13

2-D crystals of His_6_-tagged D13 were formed on lipid monolayer that contained Ni^2+^-chelating head group (DOGS-NTA-Ni). A mixture of egg L-α-phosphatidylcholine (egg-PC) and egg phosphatidyl-DL-glycerol (egg-PG) at 2∶1 (w∶w) ratio was mixed with Ni-lipid at 3∶1 (w∶w) ratio in a solvent of 80% (v/v) chloroform with 20% (v/v) methanol. 0.5 µl of this lipid solution at 0.2 mg/ml was layered on top of 15 µl of His_6_-tagged protein solution in the storage buffer partially filling a Teflon well, as described in Lévy *et al* [37]. The setup was incubated undisturbed for 1–2 hours in a Petri dish in an atmosphere of high humidity, which was provided by lining the dish with a pad of water-saturated filter paper (Whatman).

### Electron microscopy

Typically 5 µL of the sample was applied onto an EM grid covered with plastic-supported carbon film that was rendered hydrophilic by glow discharge and then negatively stained with 1.5% (w/v) uranyl acetate. For cryo-EM, 5 µL of the sample was applied to a holey grid (R2/2 Quantifoil) that was glow discharged in presence of n-amylamine. Vitrification was carried out using a Vitrobot Mark IV (FEI) at 4°C and 90–100% relative humidity. Crystals formed on a lipid monolayer were directly transferred onto a non-glow discharged holey EM grid (R1.2/1.3 Quantifoil). The grid was stained or vitrified as described above.

A Philips CM12 TEM (FEI) operating at 120 kV was used for routine examination of negatively-stained specimen where images were acquired using 1024×1024 pixel CCD camera (Bioscan, Gatan). A Philips Tecnai 12 TEM (FEI) equipped with lanthanum hexaboride (Lab_6_) gun operating at 120 kV was used to acquire images at a nominal magnification of ×52,000 that were used for further processing. Negatively stained D13 2-D crystals were tilted up to ±57° in the microscope at approximately 5° angular increments. The images were recorded on Kodak SO-163 film under low electron dose (10–20 e/Å^2^). The films were developed in D19 developer (Kodak) diluted 1∶1 (v∶v) with deionized water for 10 minutes.

### Image processing of D13 2-D crystals

Images that had minimal drift and astigmatism and yielded sharp diffraction spots were digitized using a Nikon Super Coolscan 9000 at a step size of 10.0 µm (i.e. 2549 dpi). Typically, selected 2048×2048 pixel areas from such images were processed for the 3-D reconstruction. The effects of contrast transfer function (CTF) and residual astigmatism in the digitized images were first corrected using Bsoft program package [38]. Next, these CTF-corrected images were processed using MRC program package for 2-D crystals [39] as previously described [12]. A total of 31 micrographs from two independent tilt sets were initially used for further analysis. The data from tilted crystals were merged to a common phase origin, with an imposed p6 plane group of symmetry, choosing as a reference image an untilted crystal that showed the lowest phase residual for the p6 symmetry. In all, data from 24 images that agreed best during merging contributed to the final 3-D reconstruction. The phase and amplitudes were sampled along the continuous lattice rods at intervals of z*  = 0.0067, corresponding to an arbitrarily chosen lattice dimension of c = 150 Å. Structure factor data up to maximum resolution of ∼20 Å were included in the calculation of the final 3-D density map. The handedness and vertical orientation along the z-axis of the 3-D reconstruction were determined by assigning the direction and the sign of the crystal tilt angles according to the defocus gradient along the direction perpendicular to the tilt axis. The resulting density map was displayed in UCSF Chimera [40].

### Pseudo-atomic model of D13 2-D crystals

The atomic structure of D13 was manually docked into the calculated 3-D density map of a D13 trimer. After initial manual fitting, the fitting was further optimized using ‘Fit Model in Map’ sub-routine in UCSF Chimera. Depending on the initial placement of the trimer, fitting converged to two positions related by a 60° rotation around the trimeric axis because of the pseudo-hexagonal symmetry of the trimer. The correct fit can be identified by comparison of cross-correlations between the EM density maps and the fitted X-ray structure. The correlation coefficient CC is defined as CC = <u,v>/|u||v| where u, v are vectors containing the fit map values and the corresponding interpolated reference map values respectively [40]. CC was calculated using the electron density map, where the density values were set to zero outside a broad envelope encompassing a trimer and enforcing positivity within this envelope using the USF MAVE program [41]. Cross-correlation for the correct fit was 73.5% versus 68.6% for the second best fit.

For comparison, fitting was also performed using the Situs program [42] where all six trimers of a hexameric ring in the 2-D crystal were fitted simultaneously. The final fit closely matched the best fit obtained with Chimera.

### Structure analysis and illustrations

Because of the higher resolution of the D13^D513G^ structure, this model was used for subsequent analyses and illustrations unless mentioned otherwise.

Protein interfaces and buried surfaces within the trimer and between trimers were analysed using the PISA server at European Bioinformatics Institute (EBI) [43], structural similarity were detected by PDBfold [44]. The sequence conservation was mapped onto the 3-D surface using the ConSurf server [45] with default parameters and 16 sequences of poxviruses.

Structural comparisons presented in Table S2 were produced using the secondary-structure matching (SSM) algorithm of the PDBeFold server maintained by the EBI [44]. Illustrations were prepared using PyMOL.

### Accession numbers

The atomic coordinates and structure factors of the native D13 and D13^D513G^ have been deposited in the Protein Data Bank under the accession codes 3SAQ and 3SAM respectively.

## Supporting Information

Figure S1 **Vaccinia virus morphogenesis.** Schematic representations of intermediates in the assembly of vaccinia virus. Red spikes represent the D13 protein decorating crescents, immature virions (IV) and the immature virion with nucleoid (IVN). D13 is lost in the transition to mature virions (MV) that acquire the typical brick-shape of poxviruses. Blue shapes represent the lateral bodies. Some particles bud into the Golgi compartment and gain an additional double membrane to form wrapped virions (WV). Upon exiting the cell, one of these membranes is lost to form extra-cellular virions (EV) that may remain cell-associated (CEV, not shown).
(TIF)

Figure S2 **Topology diagrams of double-barrel capsids.** The topologies of double-barrel capsid proteins of known structure are represented here. In addition, the predicted topology diagram of the mimivirus P1 capsid protein is shown and corresponds to the model presented in Figure 9E. The conserved core of double-barrel proteins is represented in blue for J1, red for J2 and cyan for the two conserved helices J1_FG_ and J2_FG_. Head domains of the poxvirus, adenovirus and mimivirus proteins are represented in gold. The topology diagram of adenovirus hexon is simplified for clarity.
(TIF)

Figure S3 **3-D reconstruction of D13 using images of tilted 2-D crystals.** (**A**) Density map corresponding to the p6 unit cell of the D13 honeycomb lattice. The magenta-cyan gradient represents the position on the Z-axis in the reconstruction and is only used for illustrative purposes. (**B**) A volume corresponding to the D13 trimer was extracted from the entire reconstruction. Scale bars represent 5 nm.
(TIF)

Figure S4 **Inter-trimer contacts in the 2-D crystalline lattice.** (**A**) Inter-trimer contacts when the X-ray structure is fitted into the 2-D honeycomb lattice of D13. Residue 513 is shown as a sphere colored in green. When this residue is mutated from Asp to Gly, D13 forms flat 2-D crystals *in vivo* rather than spherical particles. (**B**, **C**) Close-up representations of the interaction interfaces for the D_513_G and native D13 trimers respectively. The color scheme is the same as in Figure 1.
(TIF)

Figure S5 **Structural comparison of D13^D513G^ and native D13 proteins.** (**A**) Stereodiagram of superposed D13^D513G^ and D13 structures, represented in blue and yellow respectively. Residue 513 is shown as sticks. (**B**) Molecular packing in the R32 crystals of native D13. The same color scheme as Figure 1 was used. (**C**) Molecular packing in the P6_1_22 crystals of the D13^D513G^ mutant. The same color scheme as Figure 1 was used. Half of the molecules are in paler colors for clarity.
(TIF)

Table S1 **Contact areas within the D13 trimer.** * surface areas buried by the respective domains as estimated by the PISA server maintained by the EBI.
(DOC)

Table S2 **Structural similarity between the D13 scaffolding protein and double-barrel capsid proteins of large, icosahedral DNA viruses.** Statistics of structural similarities correspond to the secondary-structure matching algorithm implemented by the PDBeFold server maintained by the EBI.
(XLS)
