# Supplementary figures and images for: Membrane Remodeling by the Double-Barrel Scaffolding Protein of Poxvirus
Source: PLoS Pathog. 2011 Sep 8;7(9):e1002239. doi: 10.1371/journal.ppat.1002239 (PMC3169552; doi:10.1371/journal.ppat.1002239)

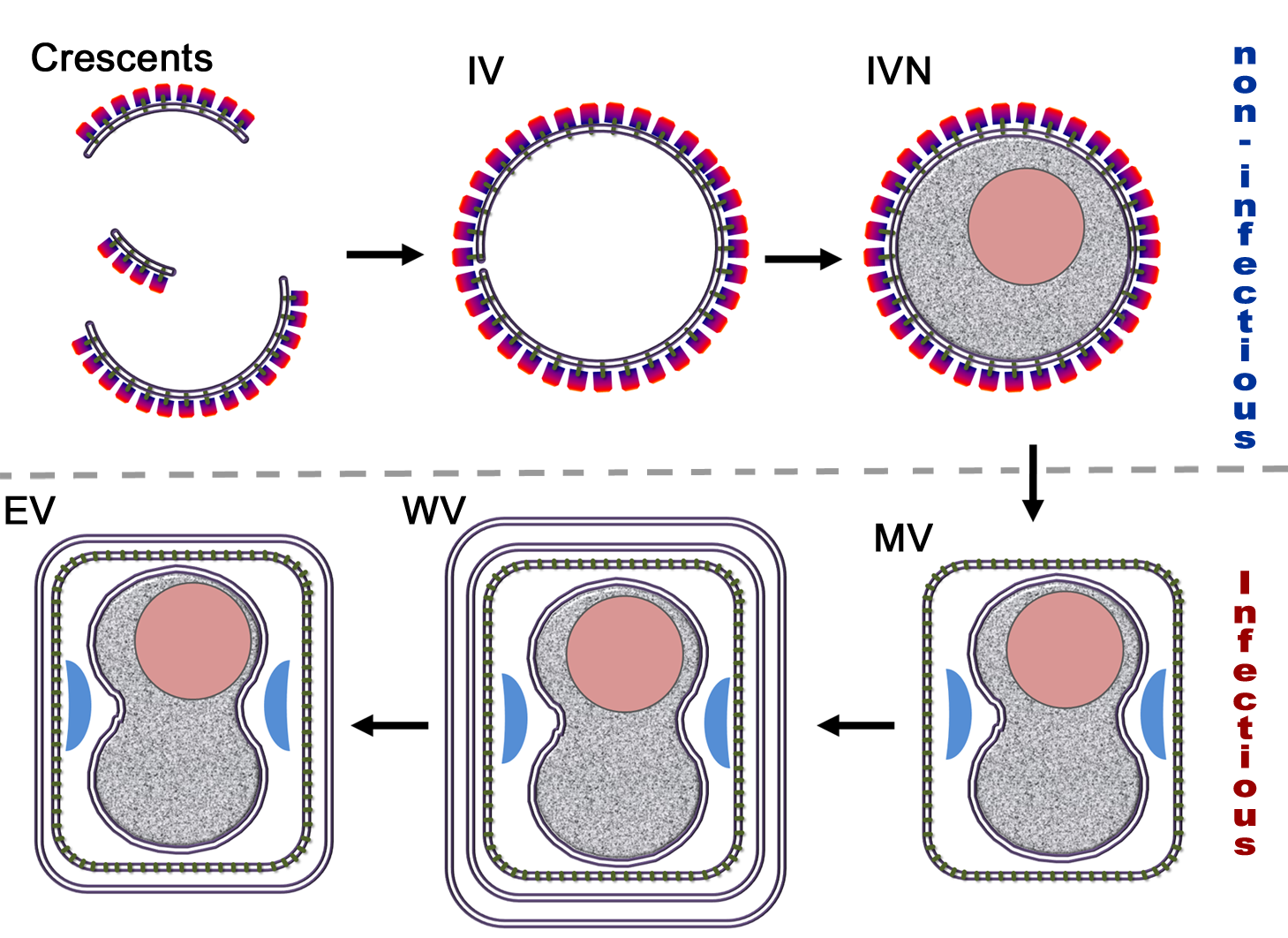

Supplement: Figure S1 — Vaccinia virus morphogenesis. Schematic representations of intermediates in the assembly of vaccinia virus. Red spikes represent the D13 protein decorating crescents, immature virions (IV) and the immature virion with nucleoid (IVN). D13 is lost in the transition to mature virions (MV) that acquire the typical brick-shape of poxviruses. Blue shapes represent the lateral bodies. Some particles bud into the Golgi compartment and gain an additional double membrane to form wrapped virions (WV). Upon exiting the cell, one of these membranes is lost to form extra-cellular virions (EV) that may remain cell-associated (CEV, not shown). (TIF) [file ppat.1002239.s001.tif]

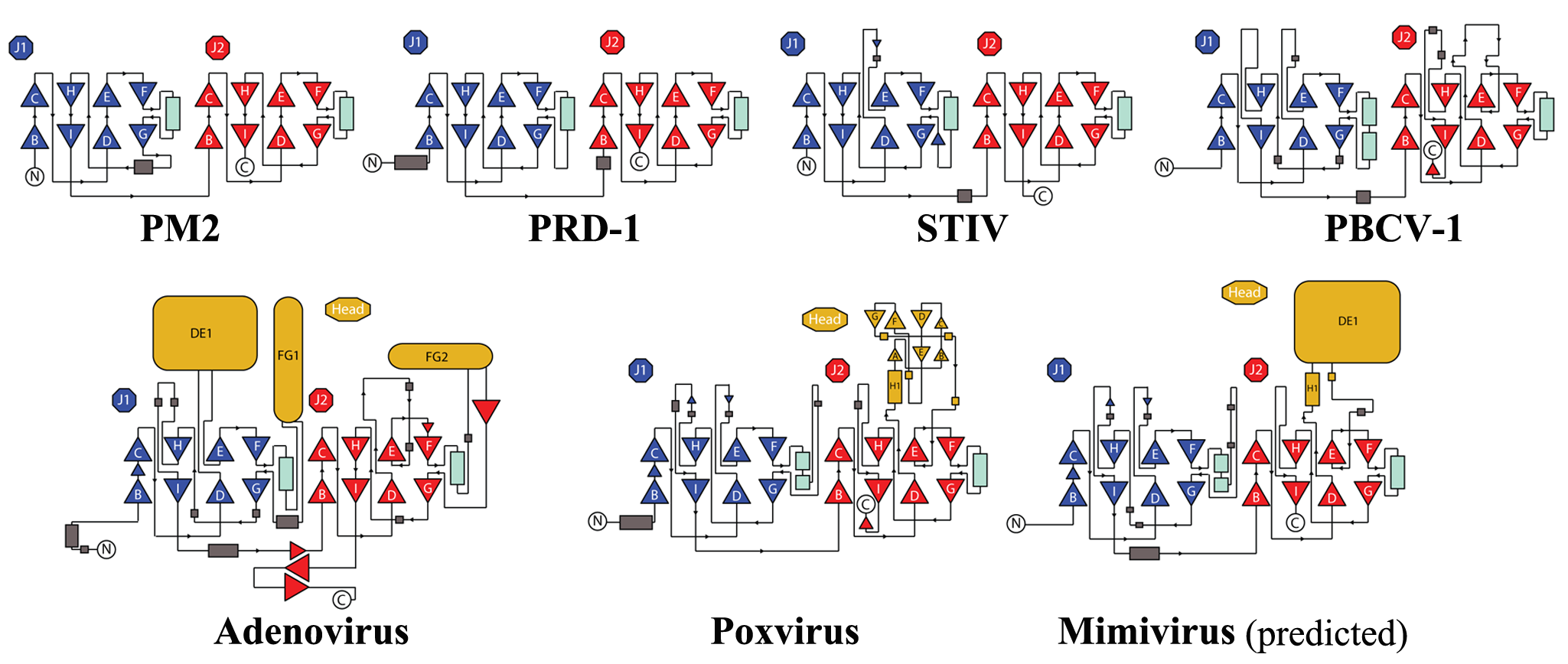

Supplement: Figure S2 — Topology diagrams of double-barrel capsids. The topologies of double-barrel capsid proteins of known structure are represented here. In addition, the predicted topology diagram of the mimivirus P1 capsid protein is shown and corresponds to the model presented in Figure 9E. The conserved core of double-barrel proteins is represented in blue for J1, red for J2 and cyan for the two conserved helices J1FG and J2FG. Head domains of the poxvirus, adenovirus and mimivirus proteins are represented in gold. The topology diagram of adenovirus hexon is simplified for clarity. (TIF) [file ppat.1002239.s002.tif]

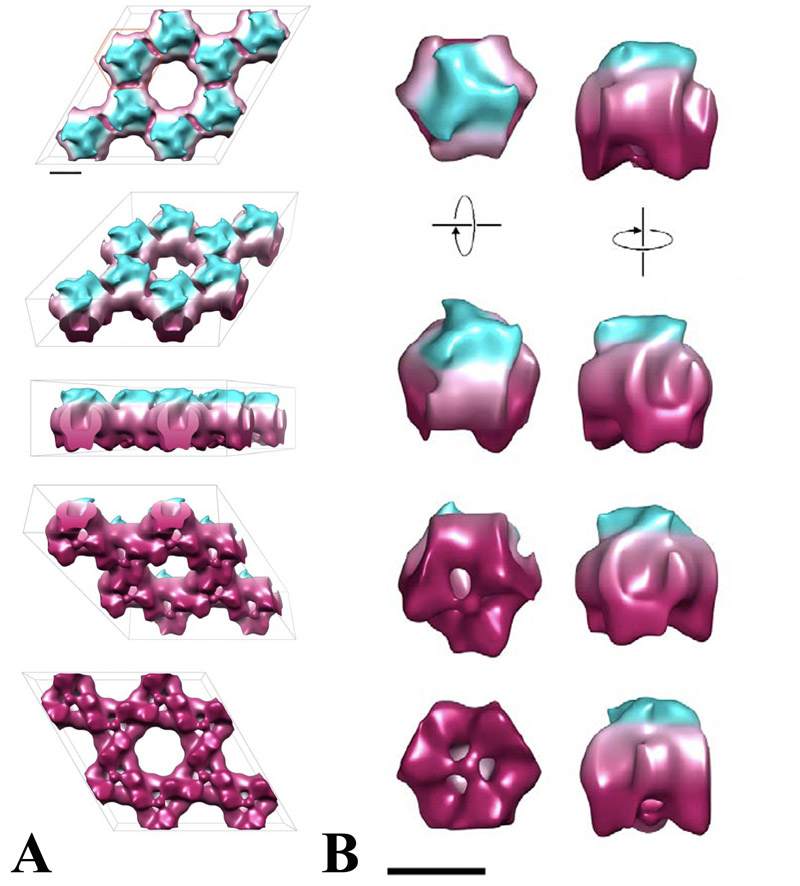

Supplement: Figure S3 — 3-D reconstruction of D13 using images of tilted 2-D crystals. (A) Density map corresponding to the p6 unit cell of the D13 honeycomb lattice. The magenta-cyan gradient represents the position on the Z-axis in the reconstruction and is only used for illustrative purposes. (B) A volume corresponding to the D13 trimer was extracted from the entire reconstruction. Scale bars represent 5 nm. (TIF) [file ppat.1002239.s003.tif]

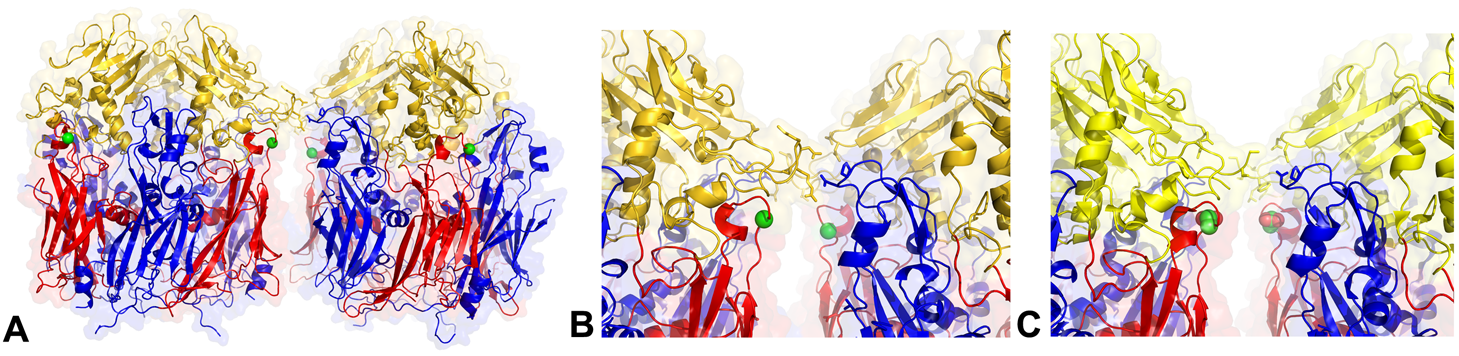

Supplement: Figure S4 — Inter-trimer contacts in the 2-D crystalline lattice. (A) Inter-trimer contacts when the X-ray structure is fitted into the 2-D honeycomb lattice of D13. Residue 513 is shown as a sphere colored in green. When this residue is mutated from Asp to Gly, D13 forms flat 2-D crystals in vivo rather than spherical particles. (B, C) Close-up representations of the interaction interfaces for the D513G and native D13 trimers respectively. The color scheme is the same as in Figure 1. (TIF) [file ppat.1002239.s004.tif]

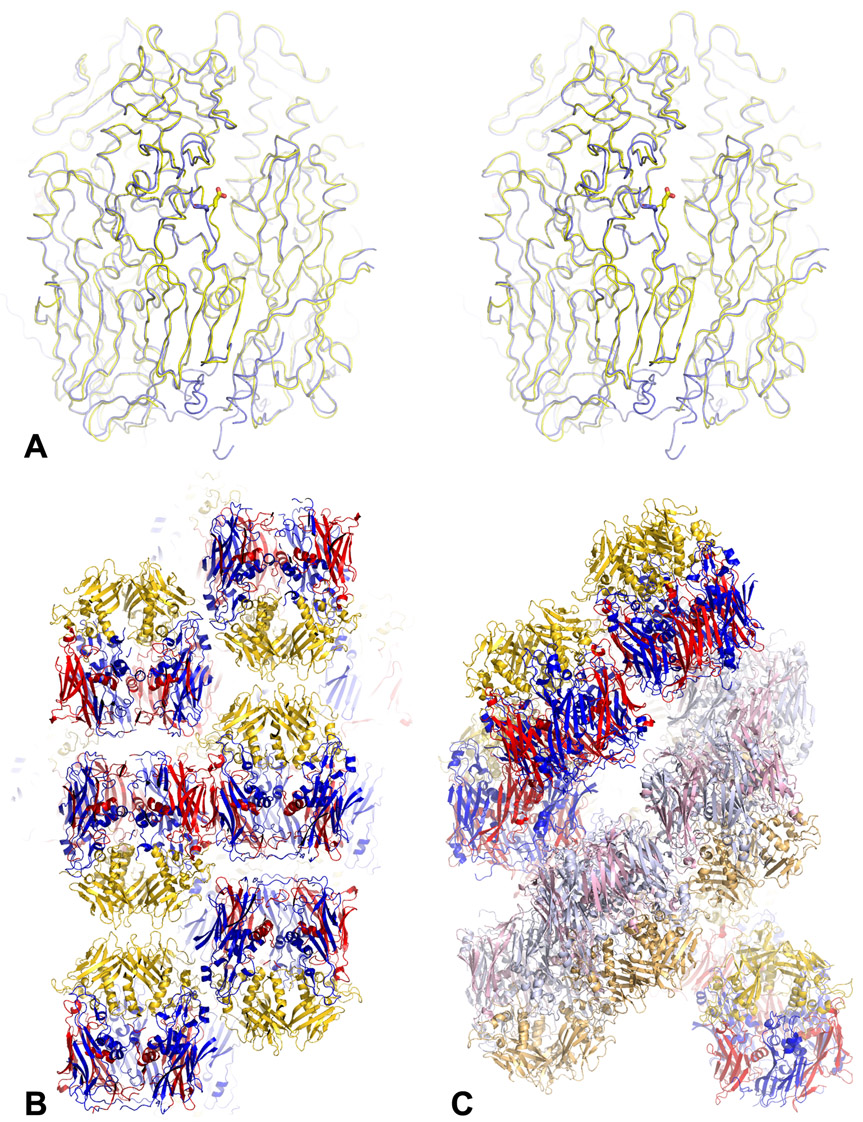

Supplement: Figure S5 — Structural comparison of D13D513G and native D13 proteins. (A) Stereodiagram of superposed D13D513G and D13 structures, represented in blue and yellow respectively. Residue 513 is shown as sticks. (B) Molecular packing in the R32 crystals of native D13. The same color scheme as Figure 1 was used. (C) Molecular packing in the P6122 crystals of the D13D513G mutant. The same color scheme as Figure 1 was used. Half of the molecules are in paler colors for clarity. (TIF) [file ppat.1002239.s005.tif]
