# Supplementary material for: Membrane Remodeling by the Double-Barrel Scaffolding Protein of Poxvirus
Source: PLoS Pathog. 2011 Sep 8;7(9):e1002239. doi: 10.1371/journal.ppat.1002239 (PMC3169552; doi:10.1371/journal.ppat.1002239)
Supplement: Table S1 — Contact areas within the D13 trimer. * surface areas buried by the respective domains as estimated by the PISA server maintained by the EBI. (DOC) [file ppat.1002239.s006.doc]

**Table S1. Contact areas within the D13 trimer**

| **Domain 1** | **Domain 2** | **Type of molecular**  **interface** | **Average***  (Å^2^) | **Individual areas***  (Å^2^) |
| --- | --- | --- | --- | --- |
| **J1** | J2 | Intra-molecular | 1084 | 1092/1090/1070 |
| **J1** | H | Intra-molecular | 851 | 855/850/849 |
| **J2** | H | Intra-molecular | 1150 | 1158/1154/1136 |
| **J1** | J2 | Inter-subunit | 1265 | 1280/1268/1246 |
| **J1** | H | Inter-subunit | None | - |
| **J2** | H | Inter-subunit | None | - |
| **H** | H | Inter-subunit | 169 | 180/165/162 |
| **J1** | J1 | Domain swap | 1004 | 1221/1117/675 |
| **J1** | J2 | Domain swap | 245 | 311/276/149 |
